# TIGER*i*: modeling and visualizing the responses to perturbation of a transcription factor network

**DOI:** 10.1186/s12859-017-1636-6

**Published:** 2017-05-31

**Authors:** Namshik Han, Harry A. Noyes, Andy Brass

**Affiliations:** 1 Gurdon Institute, University of Cambridge, Cambridge, UK; 2 School of Biological Sciences, University of Liverpool, Liverpool, UK; 3 School of Computer Science and School of Health Sciences, University of Manchester, Manchester, UK

**Keywords:** Machine Learning, Transcriptional regulatory network, Transcription factor binding site, Gene expression

## Abstract

**Background:**

Transcription factor (TF) networks play a key role in controlling the transfer of genetic information from gene to mRNA. Much progress has been made on understanding and reverse-engineering TF network topologies using a range of experimental and theoretical methodologies. Less work has focused on using these models to examine how TF networks respond to changes in the cellular environment.

**Methods:**

In this paper, we have developed a simple, pragmatic methodology, TIGER*i* (**T**ranscription-factor-activity **I**llustrator for **G**lobal **E**xplanation of **R**egulatory ***i***nteraction), to model the response of an inferred TF network to changes in cellular environment. The methodology was tested using publicly available data comparing gene expression profiles of a mouse p38α (Mapk14) knock-out line to the original wild-type.

**Results:**

Using the model, we have examined changes in the TF network resulting from the presence or absence of p38α. A part of this network was confirmed by experimental work in the original paper. Additional relationships were identified by our analysis, for example between p38α and HNF3, and between p38α and SOX9, and these are strongly supported by published evidence. FXR and MYC were also discovered in our analysis as two novel links of p38α. To provide a computational methodology to the biomedical communities that has more user-friendly interface, we also developed a standalone GUI (graphical user interface) software for TIGERi and it is freely available at https://github.com/namshik/tigeri/.

**Conclusions:**

We therefore believe that our computational approach can identify new members of networks and new interactions between members that are supported by published data but have not been integrated into the existing network models. Moreover, ones who want to analyze their own data with TIGER*i* could use the software without any command line experience. This work could therefore accelerate researches in transcriptional gene regulation in higher eukaryotes.

**Electronic supplementary material:**

The online version of this article (doi:10.1186/s12859-017-1636-6) contains supplementary material, which is available to authorized users.

## Background

Integrated functional genomics attempts to utilize the vast wealth of data produced by modern large scale genomic and post-genomic projects to understand the functions of cells and organisms [1]. The rapidly increasing amount of high throughput sequencing data makes it essential to develop new analytical tools that can systematically process and integrate those datasets. This presents both challenges and opportunities to the computer science community.

Transcription factor (TF) proteins bind to promoter elements on genomic DNA at TF binding sites (TFBS), to help control the transfer of genetic information from gene to mRNA [2]. Understanding the mechanisms underlying mRNA transcription is one of the “grand challenges” in modern biology. Experimental techniques allow direct measurement of individual gene transcription, but the contribution of multiple TFs is hard to determine [3–5]. Measuring the concentration of TF proteins and their affinity for the promoter region of genes is difficult because concentrations are low and protein-DNA interactions are subject to multiple controls, resulting in measurement artifacts [6–8]. Post transcriptional regulation compounds these difficulties because other molecules modify mRNA stability and hence the signals from the TFs [3, 9–11]. In such a complex environment, *in-silico* techniques can provide insights and hypotheses into the underlying TF regulatory activity, although they clearly have limitations.

### Reverse-engineering of TF network and TFBS information

A number of techniques are available to uncover the topology of the TF network—the networks of complex reactions and interactions in the cell that control transcript levels [12]. One strategy is to use principals of reverse-engineering and use gene expression data to infer regulatory interactions [13, 14]. Various reverse-engineering methods can reduce the dimensionality of the classic combinatorial search problem and utilize genome sequence data to enhance the sensitivity and specificity of predictions. However, they have difficulties in describing regulatory control by mechanisms other than TFs. Reverse-engineering of TF networks in the lower eukaryotes has been well developed [15–17]. However, the problems in mapping the regulatory mechanisms in cells of higher eukaryotes have made such global studies either impossible or impractical. Some recent studies have begun to address this issue [18–20], but have tended to focus only understanding which TFs bind to which genes—not looking in detail at the nature of the TF/TFBS interaction. A recent study [21] identified key biological features in transcriptional changes, however this method has difficulties in inferring the dynamics of the interactions. Furthermore, TF concentrations were not considered during the identification of the features.

**Fig. 1:**
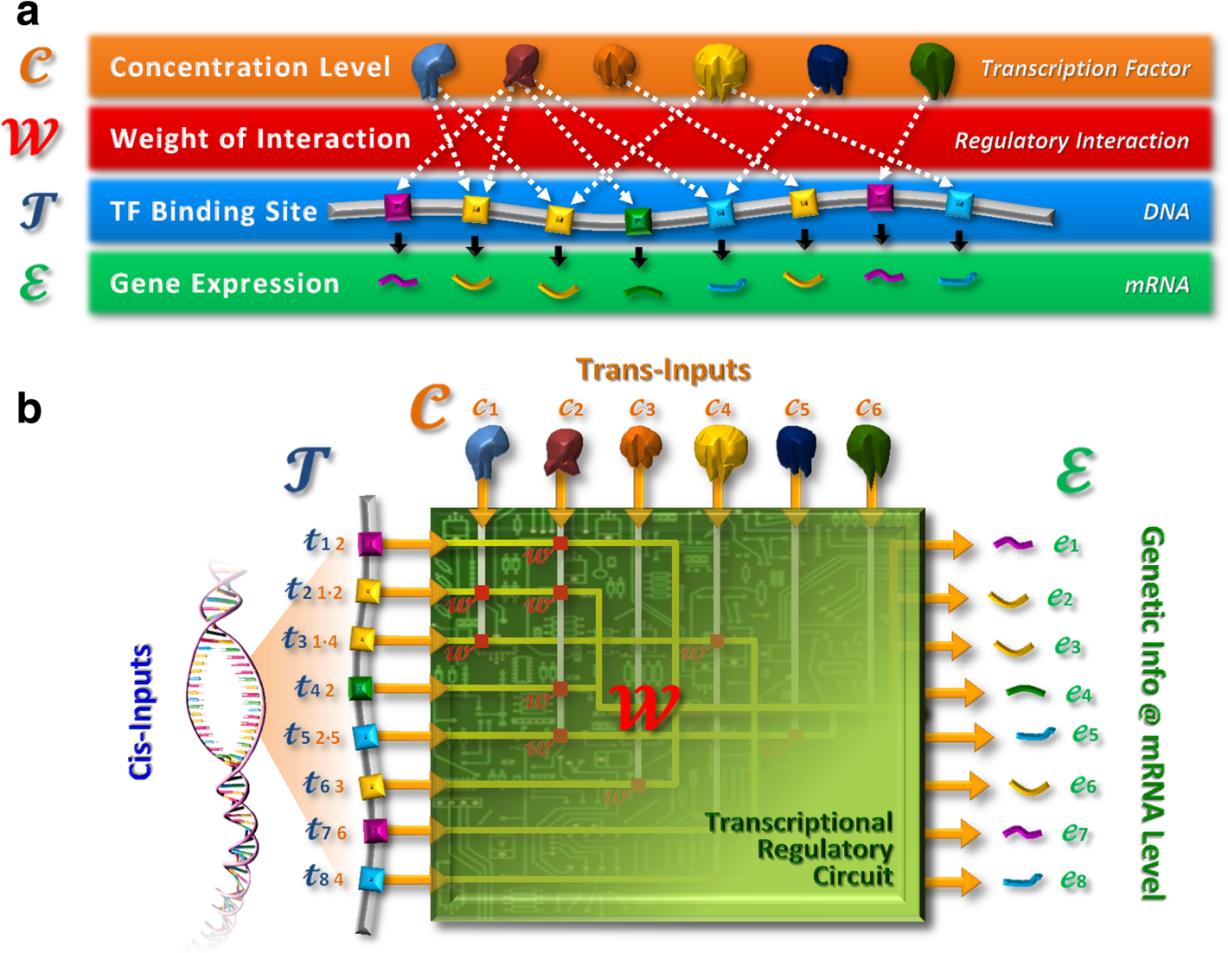
Schematic diagrams for understanding basic concepts of this study **a** The four components of transcription. The transcriptional regulators interact with their targets genes to regulate gene expression at the mRNA level. The cellular environment controls the concentration of TFs, $\mathbf{C}$. The TFs bind to specific sites close to the target genes, described in model by the connection matrix, $\mathbf{T}$. The TFs bind to their different target genes with varying strengths to regulate transcription. The strength of each of these pair-wise interactions is described by a weight matrix $\mathbf{W}$. This all finally results in the transcription of mRNA at particular concentration, ɛ. **b** A schematic of a transcriptional regulatory circuit. The circuit takes *trans-* and *cis-*inputs to transform the genetic information at mRNA level. The four components for transcription (as described above) are the key elements for the circuit

To date, various reverse-engineering methods can reduce the dimensionality of the reverse-engineering problem and utilize genome sequence data to enhance the sensitivity and specificity of predicted interactions. However, they have difficulties in describing regulatory control by mechanisms other than TFs. To address this issue TFBSs information is required to complement the gene expression data. We used a list of 132,654 TFBSs between 20,920 genes and 174 TFs that had been identified by searching an alignment of five mammal species for conserved 5’ and 3’ regions [22]. Connectivity data is notorious for high false positive rates; however, our connectivity data is robust against the problem because it extracts binding information from well conserved upstream regions. A more detailed explanation is addressed in the Methods and Results section and a schematic diagram of the connectivity data is presented in Fig. 1.

### Identifying regulation type by combining TFA and TFC analysis

Transcription factor activities (TFAs) are the intensity of the interactions between a certain transcription factor (TF) and its targets at a certain experimental point [23]. Thus, the estimated strength of TFAs between each TF and its target gene are useful to know which TF is acting on which gene at a given time point or experiment condition. However, simply knowing the regulatory activities under a single experimental condition provides limited information about the transcriptional network. To understand the mechanism of regulatory interactions, we developed a method that identifies statistically significant differences in TFAs under two different conditions. The significant differences indicate the changing level of TFAs between two conditions, so varying trends of TFAs in whole experimental process are easily detected and can be used to identify TF-specific regulatory patterns (up and down-regulation).

A highly concentrated TF induces more gene expressions rather than a lower concentrated TF. High-affinity binding sites induce the gene expressions at any level of TF concentration (TFCs), but low-affinity binding sites require high level of TF concentration for induction [24]. Thus, we might assume that TF concentration level is an important factor for investigating TFAs, and TFA investigation with considering TFC provides more reliable and accurate results closer to the complex reality of biology. To address this problem we proposed a probabilistic variational inference method to infer the concentration of each TF protein (TFC) and the regulatory intensities (TFA) of each TF and gene pair [4].

Aside from the method, there have been some notable attempts to infer TFAs based on integrating gene expression data and TFBSs information. The approaches use various well-known statistical inference techniques such as network component analysis [25], support vector machine [26], multivariate regression plus backward variable selection [27] and partial least squares [28]. However, the TFAs, which are inferred by these methods, do not contain any information on the strength and the sign of the physical interaction between a TF and its target genes. Moreover, the regulatory interactions can change easily in response to changing experimental conditions and over time. Since the methods are not fully probabilistic, they are not ideal for investigating the stochastic interactions. A linear regression based probabilistic method to model the full probability distribution of each TFA on each gene was developed [23]. The limitation of this method, however, is that it does not infer the TFAs and TFCs separately. This is a serious problem in subsequent analysis and prediction.

## Methods

### Transcription regulatory circuits and mathematical model

Transcription regulatory circuits can be thought of as having *trans-* and *cis-*inputs that are transformed into genetic information at mRNA level [29]. These circuits are a key component in the regulation of mRNA levels in the cell, and have a number of components (shown in Fig. 1): TFs, whose concentration can change, bind to TFBSs upstream of genes with a strength that is a function of the particular TF-gene interaction, to control the concentration of mRNA produced. A number of mathematical models have been developed which attempt to describe these interactions [15–17, 19, 21, 30]. For example Sanguinetti et al. [4] model the log gene expression in the form:

1 $$ \underset{\bar{\mkern6mu}}{\boldsymbol{e}}=\underset{\bar{\mkern6mu}}{\underset{\bar{\mkern6mu}}{\mathbf{\mathcal{T}}}}\underset{\bar{\mkern6mu}}{\underset{\bar{\mkern6mu}}{\mathbf{\mathcal{W}}}}\;\underset{\bar{\mkern6mu}}{\boldsymbol{c}}+\underset{\bar{\mkern6mu}}{\boldsymbol{v}} $$

Where:

i) $\underset{\bar{\mkern6mu}}{\boldsymbol{e}}$ is a set of logged gene expression measurements.
ii) $\underset{\bar{\mkern6mu}}{\underset{\bar{\mkern6mu}}{\boldsymbol{T}}}$ is a binary matrix capturing the connection topology—the specific set of TFBS upstream of genes and the TFs that bind to them. If TF ***f*** binds upstream of gene ***g*** then ${\underset{\bar{\mkern6mu}}{\underset{\bar{\mkern6mu}}{\mathbf{\mathcal{T}}}}}_{\boldsymbol{gf}}=1$.
iii) $\underset{\bar{\mkern6mu}}{\underset{\bar{\mkern6mu}}{\boldsymbol{W}}}$ is a weight matrix that captures the nature of the interaction strengths between TF-gene pairs in regulating expression of a specific gene.
iv) $\underset{\bar{\mkern6mu}}{\boldsymbol{c}}$ is the vector of concentrations of each of the TFs.
v) $\underset{\bar{\mkern6mu}}{\boldsymbol{v}}$ is a vector of independent and identically distributed variables modeling the noise in the system. The model assumes that a spherical Gaussian term could explain all noise on gene expression profiling data.

Typically, we have knowledge of $\underset{\bar{\mkern6mu}}{\boldsymbol{e}}$ (from gene expression profiling experiments, such as microarray or RNA-seq) and would like to infer the set of TFC $\underset{\bar{\mkern6mu}}{\boldsymbol{c}}$, and TFA $\underset{\bar{\mkern6mu}}{\underset{\bar{\mkern6mu}}{\boldsymbol{W}}}$ giving rise to this signal. Given $\underset{\bar{\mkern6mu}}{\underset{\bar{\mkern6mu}}{\boldsymbol{T}}}$ and $\underset{\bar{\mkern6mu}}{\boldsymbol{e}}$ Sanguinetti et al. [4] then show how it is possible to solve for $\underset{\bar{\mkern6mu}}{\boldsymbol{c}}$ and $\underset{\bar{\mkern6mu}}{\underset{\bar{\mkern6mu}}{\boldsymbol{W}}}$ using a discrete time state space model (Eq. 1) with expectation-maximization (EM) algorithm. In the model, elements of the $\underset{\bar{\mkern6mu}}{\boldsymbol{c}}$ matrix indicate the concentration level of a given TF protein (TFC) at a specific time. Elements of the $\underset{\bar{\mkern6mu}}{\underset{\bar{\mkern6mu}}{\boldsymbol{W}}}$ matrix represent the regulatory intensity (TFA) between a given TF protein and its binding affinity to its target genes. The baseline expression level is the mean vector. The measurement noise $\underset{\bar{\mkern6mu}}{\boldsymbol{v}}$ follows zero-mean i.i.d. Gaussian noise. To estimate the $\underset{\bar{\mkern6mu}}{\boldsymbol{c}}$ and $\underset{\bar{\mkern6mu}}{\underset{\bar{\mkern6mu}}{\boldsymbol{W}}}$ matrices, the model used posterior estimation of Bayer’s theorem. During this estimation, EM algorithm allowed the model to efficiently approximate the log likelihood. However, it is rare to have a complete knowledge of $\underset{\bar{\mkern6mu}}{\underset{\bar{\mkern6mu}}{\boldsymbol{T}}}$ —we simply do not know the binding sites for all TFs in a typical higher eukaryotic cell. Recent experimental techniques, such as ChIP-chip and ChIP-seq can provide useful data to help construct the connection topology $\underset{\bar{\mkern6mu}}{\underset{\bar{\mkern6mu}}{\boldsymbol{T}}}$ [31], however they have clear limitations if we are looking for a complete topology [32, 33]. A number of theoretical techniques are also available to uncover the connection topology [34–36]. The techniques generally use principals of reverse-engineering and use gene expression and genome sequence data to infer regulatory interactions.

### Gene expression data

Gene expression datasets were downloaded from Gene Expression Omnibus (accession number GSE7342 for p38α and GSE36890 for STAT5) [37, 38]. The expression profiling data of GSE7342 dataset was normalized by the robust microarray average (RMA) method. The read counts of GSE36890 dataset was normalized to the reads per kilo-base of exon per mega-base of library size (RPKM).

### Generating $\underset{\bar{\mkern6mu}}{\underset{\bar{\mkern6mu}}{\boldsymbol{T}}}$, the connection topology

In this paper we have taken a conservative strategy for generating $\underset{\bar{\mkern6mu}}{\underset{\bar{\mkern6mu}}{\boldsymbol{T}}}$ which looks at upstream region of genes that are well-conserved in multiple mammalian genomes. We used a published catalogue of common regulatory motifs that were overrepresented in gene upstream regions [22]. These motifs were identified by constructing genome-wide alignments for four mammalian species in promoter regions and 3’ UTRs relating to well-annotated genes from the RefSeq database. The same TFs were assumed to bind the same TFBSs in mice since the TFBS had been discovered in an alignment of human, mouse, rat and dog promoter regions. TFBS upstream of human 13,330 RefSeq genes were predicted. Mouse genes corresponding to the published list of human genes [22] were identified using Ensembl mouse gene annotation.

### Estimation of statistically significant changes

We were specifically interested in any TF activity that exhibits statistically significant changes between the two conditions. In particular, we are interested in changes that may be due to a change in activity of the TF, and not just in its concentration. We therefore scaled the TFA by the predicted TFC as a measure for changes in activity [24]. A joint analysis of TFA and TFC should provide more robust predictions of those TFs whose activity has changed for reasons beyond those of a simple change in concentration. To compare two different conditions, the normalized TFA by TFC in wild-type condition were subtracted by the normalized TFA in knock-out condition. We therefore determined those interactions for which:

2 $$ {\left[\left|\frac{W_{gf}}{c_f}\right|\right]}_{W T}-{\left[\left|\frac{W_{gf}}{c_f}\right|\right]}_{KO}>\boldsymbol{Cutoff} $$

The value of the ***Cutoff*** was chosen such that all differences at the 95% confidence interval were considered significant (±2 standard deviations). ±2SD limit is widely chosen as a normal limit because it fits well into two important categories: (1) confident interval and (2) testing hypothesis.

### Gene Ontology (GO) analysis

GO analysis was performed by using DAVID [39]. The sets of genes showing significant changes identified in Eq. 2 were submitted to DAVID using the default parameters in order to obtain the GO term classifications of each gene. Our computational pipeline utilized the results to investigate the functionality of genes and their regulatory TFs. The detailed methods and the result figures of GO analysis are supplied in Additional file 1: Supplementary Text and Figure S1–S3.

## Results

### Estimating the responses to perturbation of transcription networks

**Fig. 2:**
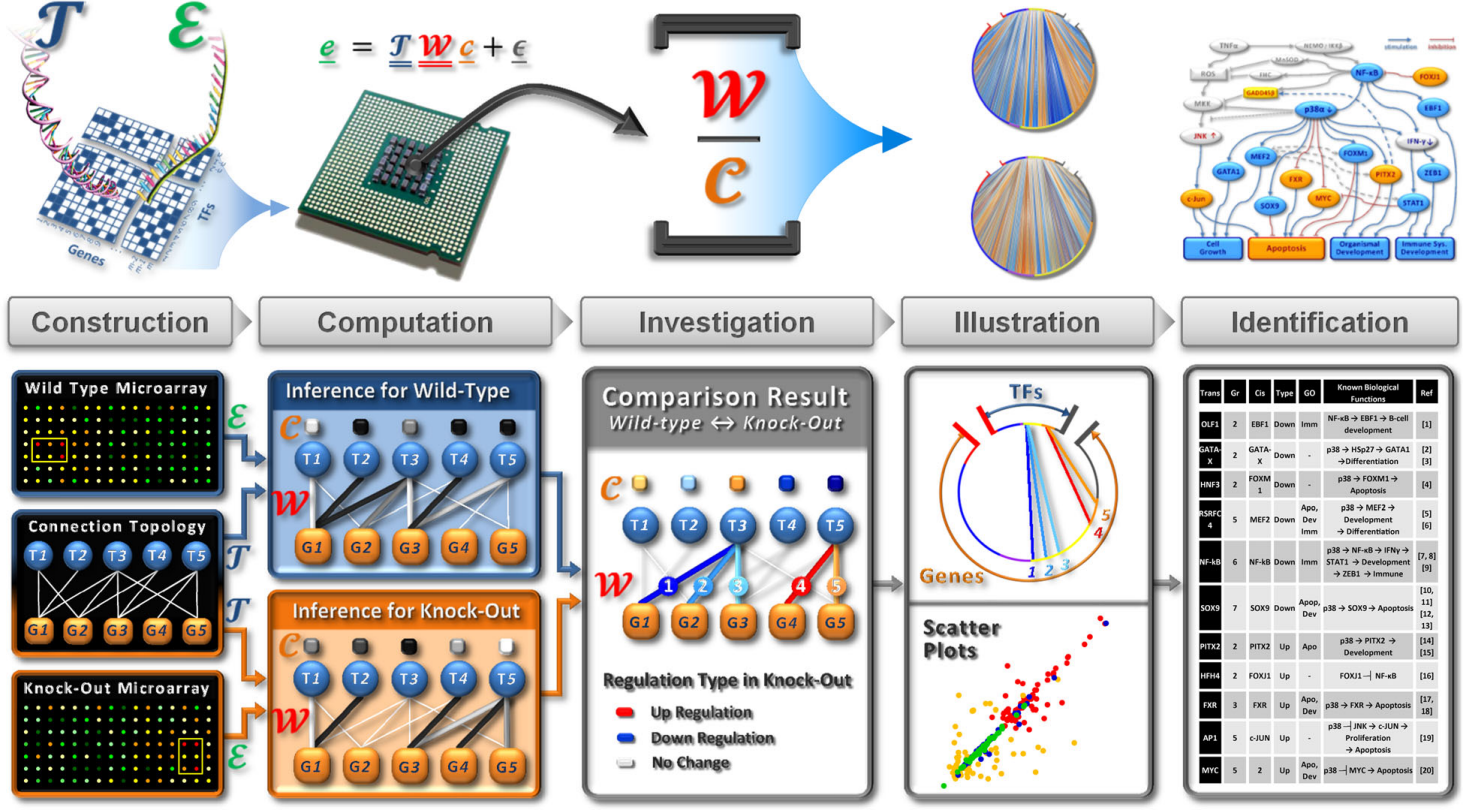
Overview of our strategy and work-flow of our computational pipeline with a plain example. Our strategy uses a computational pipeline based on a reverse-engineering technique. The pipeline takes as inputs the results of transcription (gene expression data ɛ and connectivity information $\mathbf{T}$ and outputs the sources of transcription (strengths $\mathbf{\mathcal{W}}$ and concentrations $\mathbf{\mathcal{C}}$). The pipeline is composed of five parts: Construction: RMA normalization of gene expression profiling data ɛ and a binary matrix containing connection topology $\mathbf{T}$ is constructed using by forward-engineering strategy. Computation: The gene expression profiling data and connectivity data are utilized to infer TF-gene interaction strengths $\mathbf{W}$ and TF concentration levels $\mathbf{C}$. Investigation: Once the strengths and concentrations are inferred, the actual TF activities are estimated by normalizing the strengths on the concentrations. The statistically significant changes in the TF-gene interactions strength, TF concentration levels, and TF activities are calculated. Illustration: The changes are illustrated in round limpet-like plot or in the scattered plots that shows the changes between individual TF and genes. Identification: The candidate TFs are identified, and Gene Ontology (GO) analysis are performed on the genes that are regulated by the candidate TFs. The literature is reviewed to find the supporting evidence, and the individual links between the candidate TFs and their potential biological functions are identified and summarized in a table. Based on the table, we finally construct the comprehensive TF network for p38α

We have developed a strategy which used forward-engineering to construct the connection topology (see Fig. 1, Methods, and Additional file 1: Supplementary Text and Figure S1–S3), based on a previous study of regions upstream of genes conserved in multiple mammalian genomes [22]. The structure of this network of transcriptional regulatory interactions between TFs and the genes whose transcription they control is described by a binary matrix $\boldsymbol{T}\ \in\ {\mathbf{\Re}}^{\boldsymbol{n}\times \boldsymbol{m}}$, where ***n*** is the number of TFs and ***m*** is the number of genes; An element (*i, j*) of the matrix is ‘1’ if TF ***i*** binds to the upstream control region of gene ***j***, ‘0’ otherwise. We have then employed a mathematical model to integrate the connection topology data and a gene expression dataset from a higher eukaryote in which we are interested in modeling the changes that occur in the TF network in response to a change in the cellular environment (Fig. 2). Our approach could be seen as complementary to ‘Integrative methods’, as defined in [40], as it provides a strategy for creating an approximate connection topology if more detailed information is not available. The connection topology that is being used for this analysis contains many approximations and is certainly incomplete. However, it should be noted that we are looking at the differences between the models, for example between a wild-type and knock-out state, and those differences will be in parts of the model for which we do have data.

The results of our approach provide a set of TFs and their target genes which are related by significant up- or down-regulation in transcription. It provides a clear indication of the changes in TFA and TFC of TFs that are controlling transcriptional regulatory mechanisms in response to a specific stimulus. We therefore showed an “integrated” approach for network inference, based on a forward-engineered connection topology, can produce plausible and testable hypotheses about the responses to perturbation of transcription networks in higher eukaryotes.

### Illustrating interpretable images of complex data

The visualization tools then make patterns apparent that would be difficult to detect in numerical data (Fig. 2). To distinguish regulation patterns between different experimental conditions, recognizing at a glance is important. However, computing results are formed in large numerical matrix, thus it is not only difficult to navigate through the whole matrix but also impossible to present the results in one page.

**Fig. 3:**
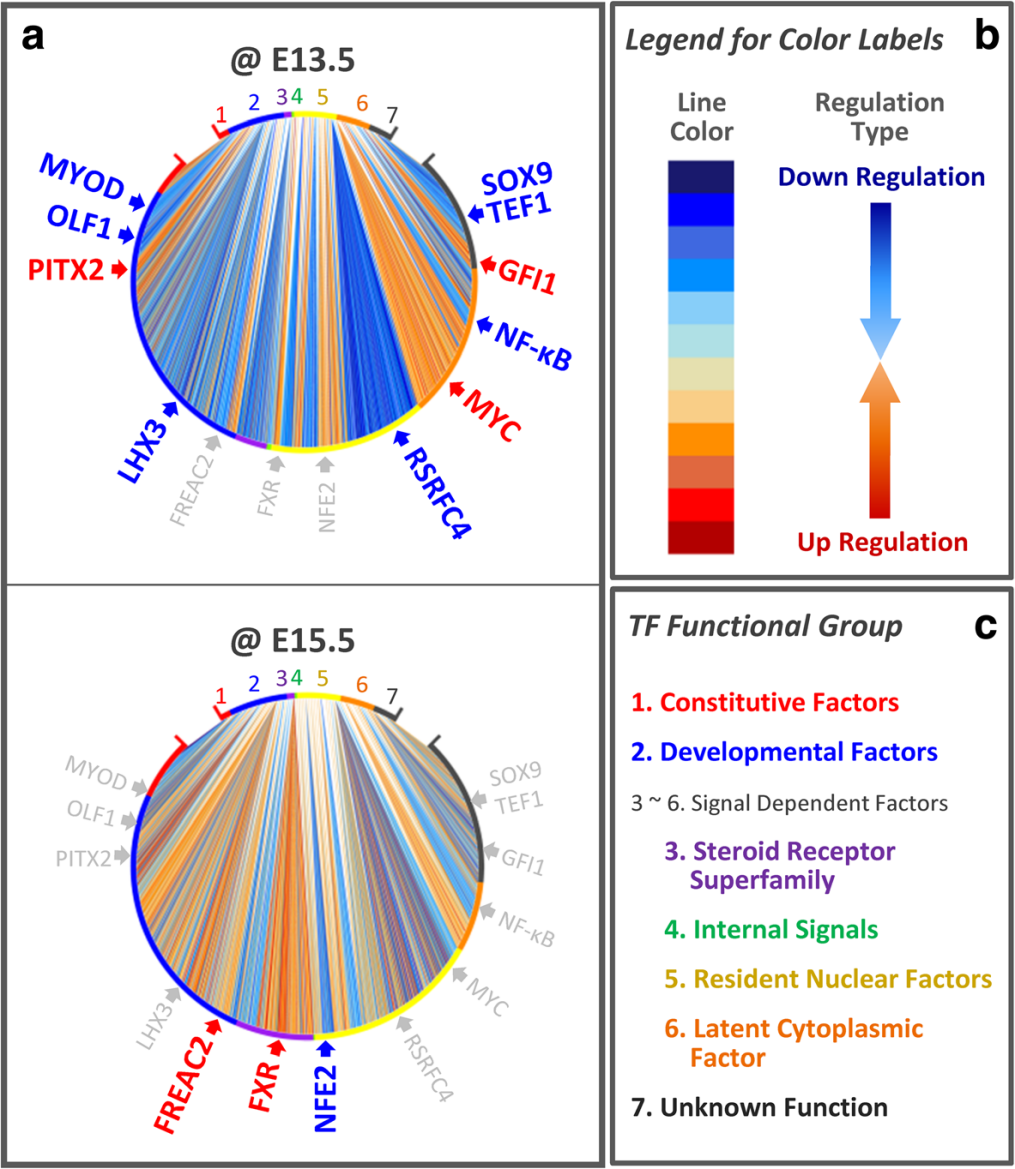
Global view of the significant changes in TF activities. Our visualization tools make it possible to distinguish specific features and trends in each condition. **a** The changes in TF activities underlying absence of p38α are presented in the limpet-like plots. In the upper part of the limpet plots, the TFs are placed in order of functional group (Fig. 3c). Genes that have at least one significant change are located in the bottom of the plots. A line presents how much the TF activity of a certain gene is changed between the wild-type mice and the knock-out mice. If a value of the change is greater than zero, it is displayed in blue indicating that the TF-gene pair has significantly higher TF activation in the wild-type mice (Down-regulation after deleting p38α); while, if the change is less than zero, it is displayed in red indicating that the pair has significantly higher TF activation in the knock-out mice (Up-regulation in deleting p38α). **b** The legend for the line color is present. **c** The perimeters of the plots are broken into different colored regions corresponding to different functional groups listed in the key

Figure 3 shows a graphical representation of the significant changes in TFA matrices $\underset{\bar{\mkern6mu}}{\underset{\bar{\mkern6mu}}{\boldsymbol{W}}}$ (***n*** *by* ***m***) and TFC vectors $\underset{\bar{\mkern6mu}}{\boldsymbol{c}}$ (***n***) obtained from this analysis. The patterns of the responses to perturbation of TF networks are readily observed in this single-shot image that presents approximately 2000 significant changes of varying TF activities on the 132,654 TFBSs after deleting p38α. In upper part of the plots, the TFs place in the functional group order. The genes, which have at least one significant interaction with TFs, locate in bottom part of the plots. A line in the plots presents a regulatory interaction (normalized TFA by TFC) between a TF and its target gene, and line color indicates a significant difference between the strengths of the regulatory interaction of two conditions. For example, we can easily find in visualized format (Fig. 3) that TF group three has distinct patterns (down-regulation at E13.5, up-regulation at E15.5) between two time points.

### Modelling the changes of transcription factor network in p38α deficient mice

The computational pipeline as highlighted in Fig. 2 was applied to a published study of the effect p38α knock-out in mouse embryos [37]. This study developed four gene expression profiling datasets (Gene Expression Omnibus, accession number GSE7342) comprising of two time points at days 13.5 and 15.5 of embryonic development (E13.5 and E15.5) for p38α knock-outs and their wild-type controls. This data set was chosen for this study as it includes experimental measurements of gene expression in the wild-type and knock-out mice and showed that p38α deficient mice have significantly different phenotype. Thus, the experimental datasets were used as positive controls for our theoretical study.

The TF-gene interaction strengths (TFAs) $\underset{\bar{\mkern6mu}}{\underset{\bar{\mkern6mu}}{\mathbf{\mathcal{W}}}}$ and TF concentration levels (TFCs) $\underset{\bar{\mkern6mu}}{\boldsymbol{c}}$ in each of these four data sets were then inferred to produce four weight matrices of TFAs:

$$ {\left[\underset{\bar{\mkern6mu}}{\underset{\bar{\mkern6mu}}{\boldsymbol{W}}}\right]}_{WT@ E13.5}, \kern0.5em {\left[\underset{\bar{\mkern6mu}}{\underset{\bar{\mkern6mu}}{\boldsymbol{W}}}\right]}_{WT@ E15.5}, \kern0.5em {\left[\underset{\bar{\mkern6mu}}{\underset{\bar{\mkern6mu}}{\boldsymbol{W}}}\right]}_{KO@ E13.5},\kern0.5em {\left[\underset{\bar{\mkern6mu}}{\underset{\bar{\mkern6mu}}{\boldsymbol{W}}}\right]}_{KO@ E15.5} $$

and four concentration vectors of TFCs:

$$ {\left[\underset{\bar{\mkern6mu}}{\boldsymbol{c}}\right]}_{WT@ E13.5},\ {\left[\underset{\bar{\mkern6mu}}{\boldsymbol{c}}\right]}_{WT@ E15.5},\ {\left[\underset{\bar{\mkern6mu}}{\boldsymbol{c}}\right]}_{KO@ E13.5},\ {\left[\underset{\bar{\mkern6mu}}{\boldsymbol{c}}\right]}_{KO@ E15.5} $$

From the TFA weight $\underset{\bar{\mkern6mu}}{\underset{\bar{\mkern6mu}}{\boldsymbol{W}}}$ and connection topology $\underset{\bar{\mkern6mu}}{\underset{\bar{\mkern6mu}}{\boldsymbol{T}}}$ matrices, the average strength of TFA, ***S***_*f*_, for each TF in the datasets was calculated:
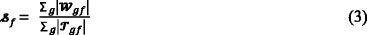


By comparing the average strengths between wild-type and knock-out mice, it is possible to see which of the TFs have significantly changed as a consequence of the removal of p38α.

**Fig. 4:**
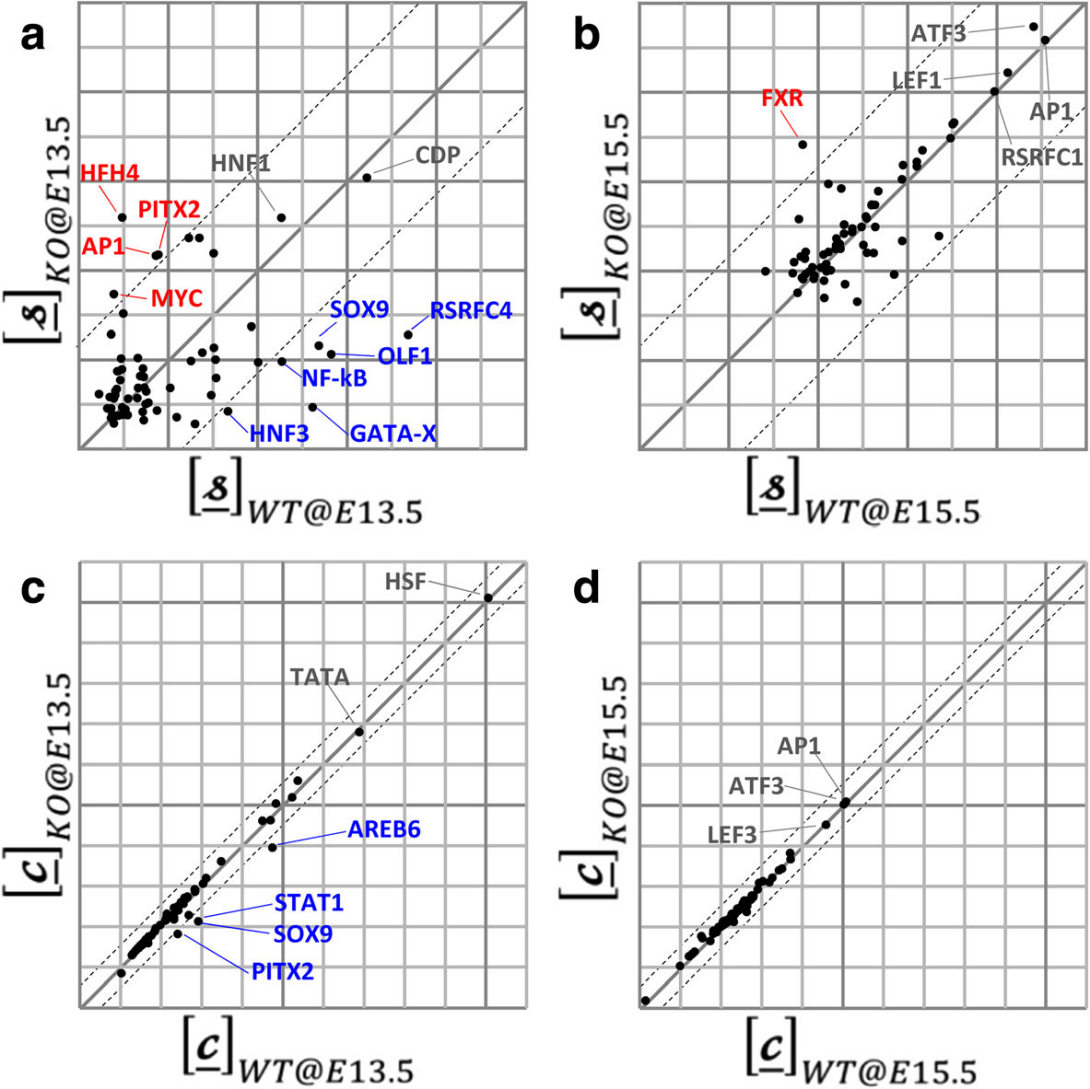
**a**, **b** The average strength of changes in TF-gene interaction $\mathbf{S}$ and TF concentration levels $\mathbf{c}$ between wild-type and knock-out mice. The figures clearly show not only which TFs have strong interaction strengths or high concentrations (*gray*-colored TFs) but also which TFs have significant changes in their interaction pattern or concentration (*blue*- or *red*-colored TFs). The dotted lines indicate the standard deviation (=2) centered on the median value of the straight lines. (figure a for time point E13.5 and figure b for time point E15.5). Five TFs (shown in *red*) interact particularly strongly with their target genes in the p38α knout-mice. In contrast, six TFs (shown in *blue*) interact less-strongly in the p38α knock-out than wild-type. **c**, **d** The TF concentration levels 풸 of wild-type and knock-out mice. TF concentration levels are plotted. The strongest signal was observed in E13.5, only. Deleting p38α induces a down regulation of AREB6, PITX2, STAT1 and SOX9

Figure 4a, b show the changes in TFA strengths  between wild-type and knock-out mice at E13.5 and E15.5. It can be seen that a number of TFs show a significant signal (>2 s.d.) in this data. These are shown with more detail in Table 1. Figure 4c, d show the inferred TFCs $\underset{\bar{\mkern6mu}}{\boldsymbol{c}}$ obtained for the E13.5 and E15.5 time points. Again, from this graph it is possible to see that a number of TFs appear to be responding to the p38α status. These are shown in more detail in Table 2.

**Table 1:** TFs showing significant changes in interaction strength between wild-type and knock-out mice

*Trans* name	TF group	*Cis* name	Regul-ation type	GO analy-sis	Known biological functions	Ref.
OLF1	1.Dev	EBF1	Down	Imm	NF-κB → **EBF1** → B-cell development	[52]
GATA-X	1. Dev	GATA1 ~6	Down	-	p38 → HSp27 → **GATA1** → Differentiation → **GATA1** → IL9 → Asthma	[53, 54]
HNF3	1. Dev	FOXM1	Down	-	p38 → **FOXM1** → Apoptosis	[45]
RSRFC4	5. Res	MEF2	Down	Apo, Dev Imm	p38 → **MEF2** → Development → Differentiation	[46, 55]
NF-κB	6. Lat	NF-κB	Down	Imm	p38 → **NF-κB** → IFNγ → STAT1 → Development → ZEB1 → Immune	[56–58]
SOX9	7. Unk	SOX9	Down	Apop, Dev	p38 → **SOX9** → Apoptosis	[59–62]
PITX2	1. Dev	PITX2	Up	Apo	p38 → **PITX2** → Development → Apoptosis	[63, 64]
HFH4	1. Dev	FOXJ1	Up	-	**FOXJ1** ─┤ NF-κB	[65]
FXR	3. Ste	FXR	Up	Apo, Dev	p38 → **FXR** → Apoptosis	[66, 67]
AP1	5. Res	c-JUN	Up	-	p38 ─┤JNK → **c-JUN** → Proliferation → Apoptosis	[37]
MYC	5. Res	MYC	Up	Apo, Dev	p38 ─┤**MYC** → Apoptosis	[68]

The TFs predicted to have significantly different behaviors between the wild-type and knock-out mice. “Trans Name”—the official gene symbol of the TF. “Cis Name”—the name given to the binding site of the TF. TFs were characterized into different functional groupings (see Fig. 3c for details: Dev—cell-type specific developmental TFs, Res—signal dependent resident nuclear factors, Lat—signal dependent latent cytoplasmic factors, Ste—signal dependent steroid receptor group, Unk—unknown). “Regulation Type”, the way in which the TF regulates its target genes in the absence of p38α. “GO Analysis”, provides more functional classification for the identified TFs (see Methods and Additional file 1: Figure S1 to S3 for details). The abbreviations of the GO terms are: Apo, Apoptosis; Dev, Developmental Process; Imm, Immune System Development. “Known Biological Functions” summarizes the findings from the recent biological literature as shown in the “References”

**Fig. 5:**
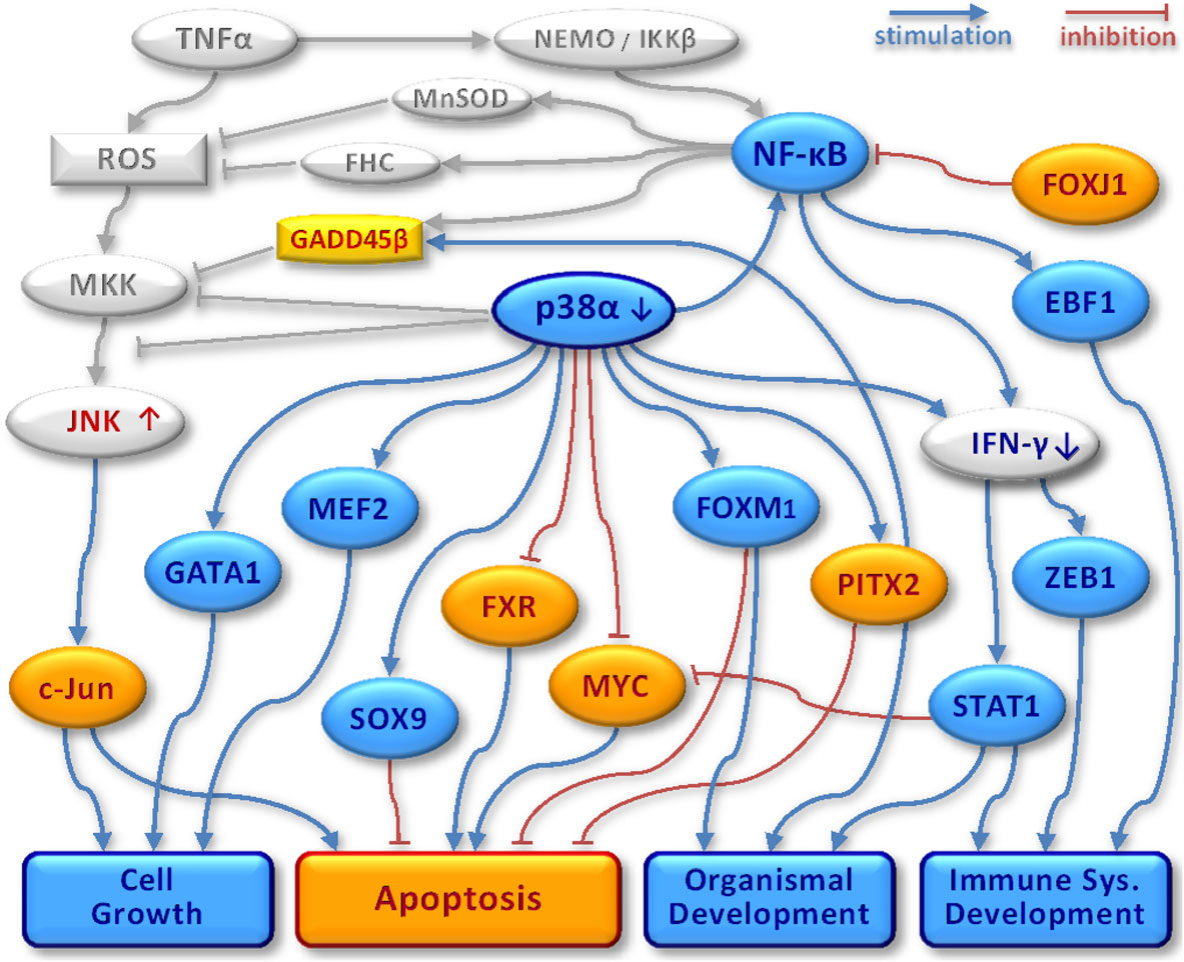
A comprehensive transcriptional regulatory network for the p38α. The TFs depicted in gray is already known to p38α [41, 51]. The TFs identified in our analysis were then added to the figure and colored blue if their activity was down regulated in the absence of p38α, and colored orange if their activity was up regulated in the absence of p38α. Lines in the figure represent interactions that are known in the literature (listed in more detail in Tables 1 and 2)

The boldface ones are the main node in Fig. 5

**Table 2:** TFs showing significant changes in concentration between wild-type and knock-out mice

*Trans* name	TF group	*Cis* name	Level	GO analy-sis	Known biological functions	Ref.
AREB6	1.Dev	ZEB1	Down	-	p38 → IFNγ → **ZEB1** → Immune	[58]
PITX2	1.Dev	PITX2	Down	Apo	p38 → **PITX2** → Development → Apoptosis	[63, 64]
STAT1	6. Lat	STAT1	Down	-	**STAT1** → Development → Immune	[57, 69]
SOX9	7. Unk	SOX9	Down	Apo, Dev	p38 → **SOX9** → Apoptosis	[59–62]

TFs changing their concentration levels significantly between wild-type and knock-out mice. “Level”, the changes of TF concentration level in the absence of p38α. Other column headings and abbreviations are the same as those in Table 1

The boldface ones are the main node in Fig. 5

### Transcriptional regulatory network for p38α

Gene Ontology (GO) analysis on the target genes of the TFs with strongly changed activity showed enrichment for three GO terms and provided insight into the functional role of the TFs (see Methods, Tables 1 and 2, and Additional file 1: Supplementary Text and Figure S1–S3). The three GO terms are the regulations of the apoptosis (programmed cell death), the downward spiral of the developmental process, and the immune system development. The JNK-c-Jun pathway stimulates the apoptosis, and the I-kB kinase/NF-kB cascade acts as a suppressor of the JNK-c-Jun pathway [41]. Inhibition of p38α MAPK retards another JNK-c-Jun pathway inhibitor NF-kB cascade, but promotes JNK-c-Jun pathway which induces the apoptosis by expressing the Bcl2 protein family [20, 42]. On the other hand, developmental process related genes are down-regulated in the p38α knock-out mice. The study of p38α MAPK [37] reported that the p38α knock-out mice die within days after birth. We do not have enough gene expression profiling data (either other time points in the embryonic period or postnatal period) to investigate TFAs in whole developmental process of the p38α knock-out mice; we cannot confirm but suppose that it might be the reason of the death of the knock-out mice. Further, the genes interact with the TFs which are reported as crucial TFs in the developmental process and the immune system development. Our results are therefore in broad accordance with the experimentally validated results, so it confirmed that our pipeline produces reliable results.

Combining the data in Tables 1 and 2 with those obtained from the literature it is possible to build a putative model for the effects of p38α knock-out (Fig. 5). This figure shows TFs with a strong response in our analysis as nodes, with links that demonstrate regulatory interactions between them. The TF network therefore comprehensively shows the biological consequences of p38α knock-out at transcriptional level.

## Discussion

We have developed a novel strategy for discovering changes in transcriptional regulatory networks of higher eukaryotes. It integrates methods for inferring TF-gene interaction strengths (TFAs) and TF concentration levels (TFCs); identifying statistically significant changes in TFAs and TFCs; analyzing the changes; classifying TFs into functional groups; and visualizing the changes. To our knowledge, this is the first ensemble approach for characterizing the transcriptional function of TF proteins and their target genes in higher eukaryotes. Reverse-engineering of TF networks has been well developed in the lower eukaryotes [15, 17]. However, the problems in mapping the regulatory mechanisms in cells of higher eukaryotes have made such global studies either impossible or impractical. Some recent studies have begun to address this issue [16, 19, 30], but have tended to focus only on understanding which TFs bind to which genes—not looking in detail at the nature of the TF-gene interaction. Other studies [5, 21] identified key biological features in transcriptional changes, however the methods have difficulties in inferring the dynamics of the interactions. A recent review [40] has categorized techniques for network inference and listed their limitations.

We validated our computational pipeline using the p38α gene expression profiling data and our connectivity data. The study of p38α MAPK [37] used various experimental methods including a gene expression profiling analysis to show that p38α negatively regulates cell proliferation by antagonizing the JNK-c-Jun pathway. We utilized the published gene expression profiling dataset from their study, to demonstrate that our computational pipeline is able to infer from the gene expression profiling data the same *in-silico* conclusions that the authors obtained from their in-vitro experiments. Therefore, our analysis focused on the JNK-c-Jun pathway to validate the accuracy, robustness and reliability of our strategy. The results are consistent with the experimentally validated inhibitory effect of p38α on transcriptional networks [37]. Their published data confirmed that the most important TF involved in the response to the knock-out was c-Jun, with a clear change observed in both its activation and concentration. In our theoretical work, we also showed a significant change in TFA of c-Jun, but we did not see any corresponding change in the predicted TFC, which is disappointing.

The p38α MAPK is one of many signal transduction pathways and works in both cell-type specific and cell-context specific manner. It plays a pivotal role in converting extra-cellular signal into a wide range of cellular response [43]. We classified a set of TFs that responded to the deletion of p38α into functional groups (Tables 1 and 2, Fig. 3c), that are either developmental factors (group 2) or extra-cellular signal dependent factors (group 3). Developmental factors are also dependent on extra-cellular signals because cells may require such signals to generate developmental factors [44]. In Fig. 3a, it can be seen that the main factors that responded to the knock-out are the extra-cellular signal dependent factors. None of the TFs that significantly respond in the knock-out are constitutive factors. Our results are consistent with recent publications on the JNK-c-Jun pathway (see citations in Tables 1 and 2).

Our analyses generated a comprehensive transcriptional regulatory network for p38α. The network and a detailed description are shown in Fig. 5. The nodes in the graph were generated from our analysis of responding TFs. The edges in this network were derived from the literature or GO analysis (citations in Tables 1 and 2). The edges or links in the network of p38α regulated TFs have mostly been previously reported, but none of the reports had integrated all these p38α related TFs into a single comprehensive network diagram. Together these results predict a set of TFs that are in some way regulated by p38α, a set somewhat larger than that identified in the original paper. For example, we predict that Foxm1 (HNF3) responds to the p38α status. Recent papers, published since the original study, provide some support for this hypothesis [45, 46]. Most parts of the network are reported in numerous biological studies. However, our network reveals novel links such as p38α─FXR and p38α─MYC. The inferred links are supported by direct experimental evidence so validating the approach, but that in addition novel links have been proposed that are now testable.

The data shown in Tables 1 and 2 and visualized in Fig. 5, provide evidence that the methodology described in this paper is capable of generating plausible hypotheses about linkage between p38α and a range of different TFs. The hypotheses presented in this table have been generated solely from our input data (the connection topology data and gene expression profiling data), but are well-supported by the literature. The methodology has therefore demonstrated that it can produce plausible and testable hypotheses, even if the specific details of those interactions may not be completely accurate. This is not surprising given the fact that we only have an incomplete model of the transcription process. Any *in-silico* techniques which uses predicted TF/TFBSs interactions can provide only a limited view of the complete complexity of transcription control due to the nature of the binding between the TF and the TFBS and the complex effect of gene expression on the TFBS—for example dependent on the epigenetic factors, such as the pattern of histones or DNA methylation at the binding site—as well as the state and concentration of the TF itself. Analysis is complicated by the fact that there are other processes in the cell that act to control mRNA concentration. Such as the rate of RNAi regulated mRNA degradation [9, 10] or susceptibility to attack by RNAses [3, 11]. TFBS can hidden by histones [7, 8], or made more accessible by genomic uncoiling [6]. Furthermore, most TF binding may be cell or species specific not all sites are functional even if occupied, and many functional sites have low levels of conservation [47]. This rather undermines the commonly accepted assumption that TFBSs can be discovered by conservation [22]. However although the exact binding sites may not be conserved the set of TFs that bind a gene somewhere probably is.

**Fig. 6:**
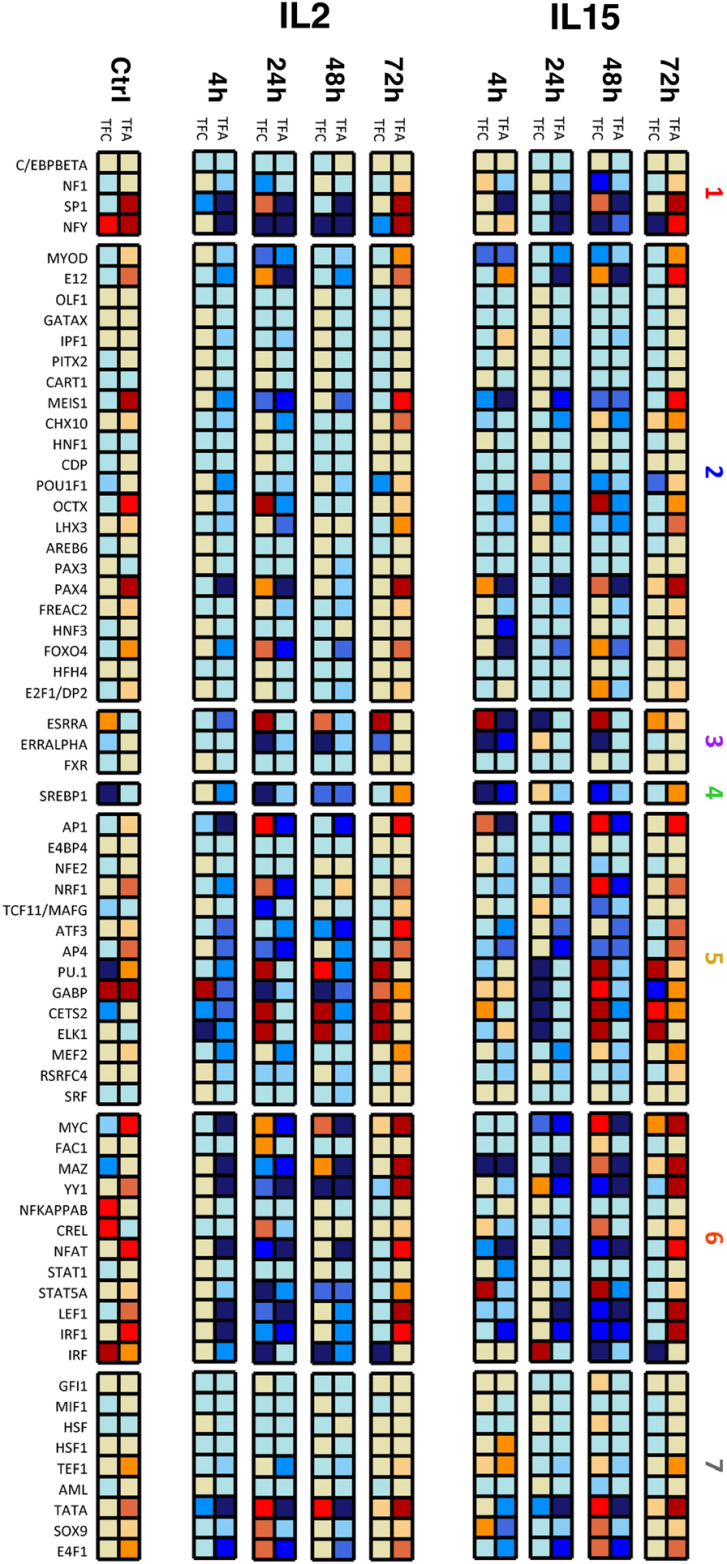
TFA and TFC changes in STAT5-tetramer deficient mice. TFA and TFC of 65 TFs were estimated from IL2- and IL5-induced RNA-seq datasets and compared between wild-type and STAT5-tetramer deficient mice. Thus, TFA or TFC of a given TF is shown in *red* color if it is higher in STAT5-tetramer deficient mice than wild-type mice. If the level of TFA or TFC is higher, the color is darker. The numbers in right-side of heat-map indicates TF functional group (please see legend in Fig. 3c)

p38α deficient mice showed significantly different phenotype which indicates its role is critical. The p38α study also provided the gene expression profiling dataset of wild-type mice as well as p38α deficient mice, so that we could apply our pipeline on the dataset to investigate TFAs and TFCs. It allowed us to directly compare our *in-silico* results to the experimental in-vitro results, and it validated our findings. However, the experiment was done on two time-points that could limit our validation. Thus, we tested our pipeline on a larger dataset from a recent STAT5 transcription factor study [38] which is consisted of 18 samples in five time-points. This study showed the critical role of STAT5-tetramer in immune system. To do this, the authors made STAT5-tetramer deficient mice by generating STAT5A-STAT5B double-knockin mice. Interleukin 2 (IL2) and IL15 are two of well-known upstream regulators of STAT5A-STATB, so they measured IL2- and IL15-induced gene expression profiling in both wild-type mice and STAT5-tetramer deficient mice. We downloaded the RNA-seq gene expression dataset from this study and analyzed with our pipeline. TF activities were decreased in STAT5-tetramer deficient mice (both IL2- and IL15-induced), particularly at 4, 24, 48 h (Fig. 6). This general trend is well-corresponded to the experimental findings as the author reported IL2- and IL15-induced gene expression were both down-regulated in STAT5-tetramer deficient mice. However, most TFs were re-activated at the last time-points which is also exactly same observation in the experimental result. In particular, STAT5A is in our TF list so we closely investigated its activation patterns. STAT5A showed weak activities and concentration level at control sample, but it dramatically de-activated at 4, 24, 48 h and then re-activated at 72 h. More interestingly, all TFs in cytoplasmic factor group (marked as number 6 in Fig. 6) including STAT5A have same activation patterns with STAT5. Moreover, there are a few interesting TFs which are not classified as cytoplasmic factor but also followed same up- and down-regulated patterns with STAT5 (e.g. SP1, NFY, E12, MEIS1, PAX4, AP1, NRF1, TCF11, AP4, GABP, TATA, E4F1). We considered these are new findings and could lead new insight and testable hypotheses.

## Conclusions

Our objective was to develop an effective computational pipeline which produces reliable and explicit models of transcriptional regulatory networks. Even though the TFBS information is incomplete due to the difficulties in identifying them, our pipeline predicts new biological hypotheses on a genome-wide scale by combining TFBS and gene expression information. TIGERi is publicly available as a stand-alone GUI software, so ones who have their own gene expression profiling data could easily use the TIGERi software to analyze their data on their fingertips. It would facilitate transcriptional gene regulation researches in the biomedical community.

Our approach can be applied to other gene expression datasets to provide a display of the transcriptional regulatory networks and identify novel candidate genes and TFs underlying specific phenotypes. For example, our methodology has been successfully applied to three recent studies [48–50]. The pipeline would be particularly valuable if it were run on large-scale multi-time point genomic data. It is also the case that we might expect the method to become increasingly predictive with improved connection topologies created from large scale experimentally validated TF/TFBS datasets as opposed to those generated from simple conservation data.

## Additional file

Additional file 1: Supplementary Text and Figures. (DOC 7227 kb)
