# Supplementary material for: TIGERi: modeling and visualizing the responses to perturbation of a transcription factor network
Source: BMC Bioinformatics. 2017 May 31;18(Suppl 7):260. doi: 10.1186/s12859-017-1636-6 (PMC5471961; doi:10.1186/s12859-017-1636-6)

**Supplementary Text**

**Connection Topology Data.** The connection topology data is shown in Table S1. The first three columns provide information on the transcription as provided in .

TF name: the known name of the TF

Cluster number: TF protein identifier from .

Motif: The conserved motif defining the cluster.

The remaining columns show information on the TF’s target genes with their official gene symbol, descriptions, and RefSeq IDs that are followed by human (Hs) and mouse (Mm) genome coordinates and Ensembl IDs, respectively.

**Gene Ontology (GO) Analysis.** To investigate further the biological functionality of TFs, GO analysis was performed. The significantly differently expressed genes were identified by Eq. 3. The two sets of Affymetrix identifiers for the significantly up/down regulated genes were then inputted into DAVID version 6 with default parameter settings. The outputs of DAVID were GO terms and the lists of contributed genes for the GO terms with enrichment scores. Next, the genes for each GO term were mapped to the TFs regulating the genes. The mapping results provided the contribution levels of each TF to the GO terms, thus the results hinted at biological functionality of TFs that could provide better understanding of the alternations of transcriptional regulatory mechanism to perturbation.

The contribution levels of each TF are presented in figure S1 to S3. Those three images are for apoptosis, immune system developmental process, and organ developmental process, respectively. The three GO terms have the highest enrichment scores among the GO analysis results. Each image is composed of two parts, (a) for TF activities at E13.5 and (b) for TF activities at E15.5. The dotted line indicates standard deviation 2, thus each figure shows the TFs that are highly involved to each GO term. For instance, PITX2 and MYC are positively correlated TFs to apoptosis at E13.5 (figure S1 a), however MYOD, LHX3, RSRFC4, TEF1, and SOX9 are negatively correlated TFs to apoptosis.

**References**

1. Xie X, Lu J, Kulbokas EJ, Golub TR, Mootha V, KLindblad-Toh V, Lander ES, Kellis M**: Systematic discovery of regulatory motifs in human promoters and 3' UTRs by comparison of several mamma**ls*. Natur*e 2005**, 4**34:338-345.

**Supplementary Figures**

Fig S1. TF activities involving with apoptosis-related genes. (a) TF activities at E13.5 (b) TF activities at E15.5


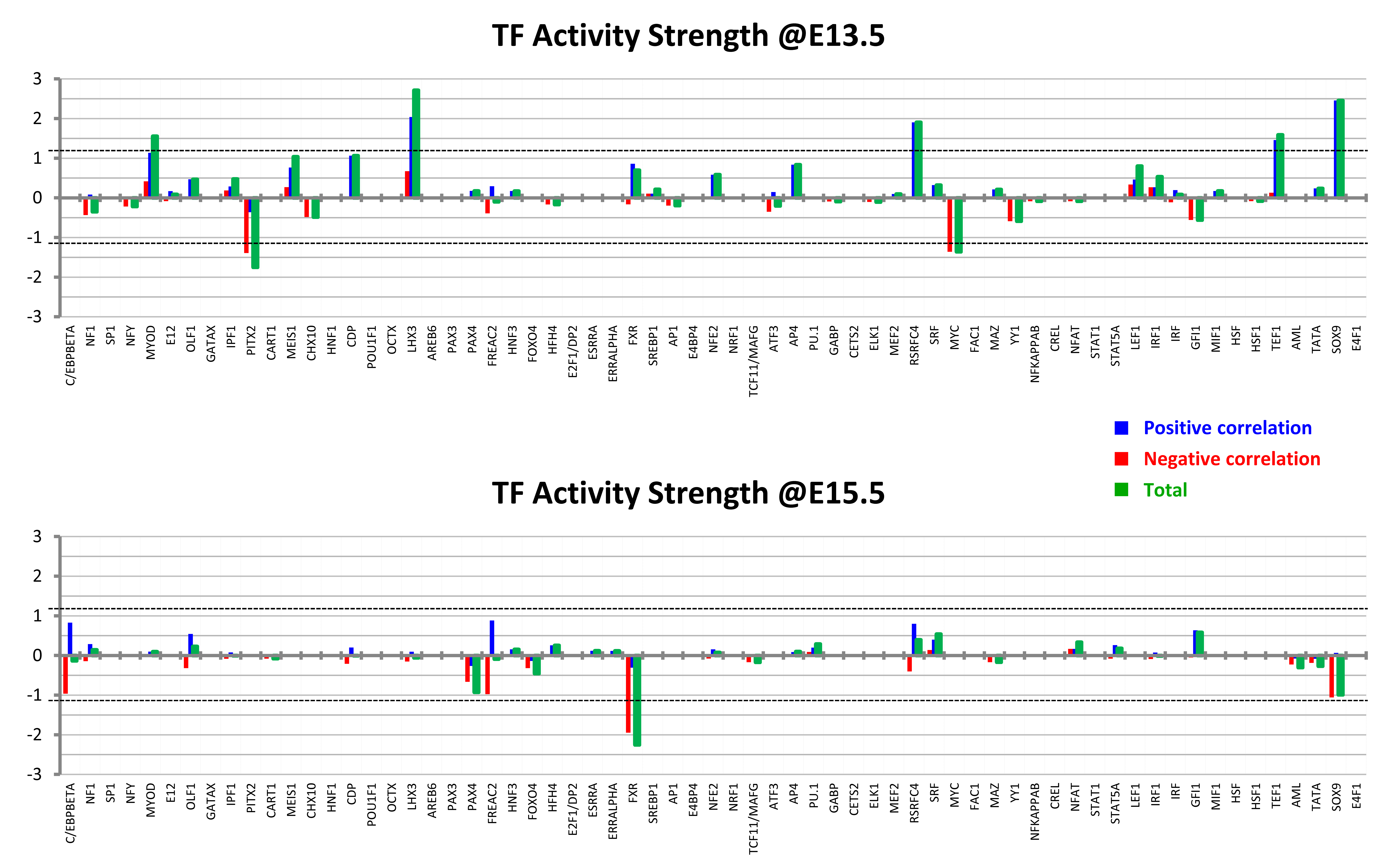


Fig S2. TF activities involving with immune system development-related genes. (a) TF activities at E13.5 (b) TF activities at E15.5


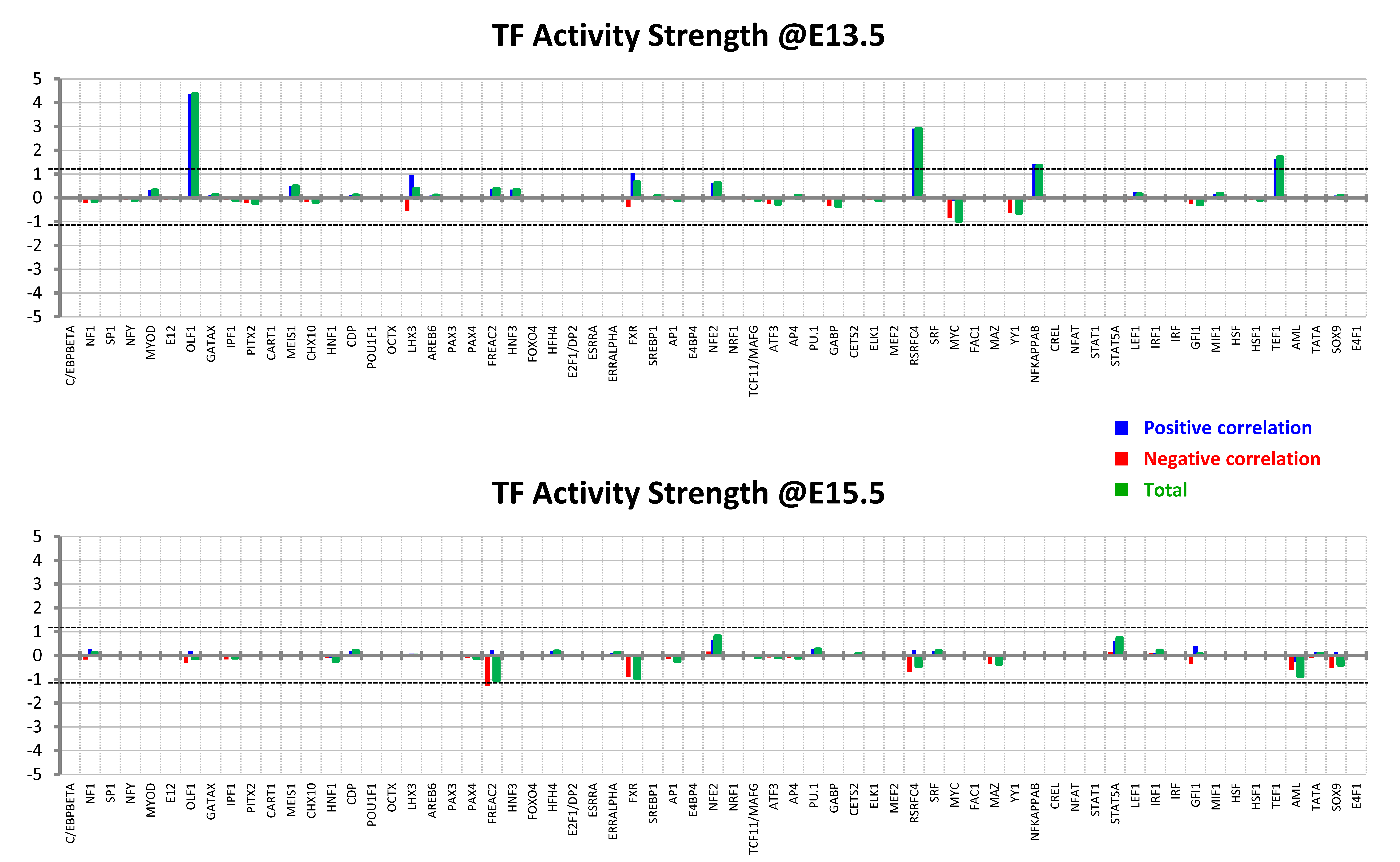


Fig S3. TF activities involving with developmental process-related genes. (a) TF activities at E13.5 (b) TF activities at E15.5


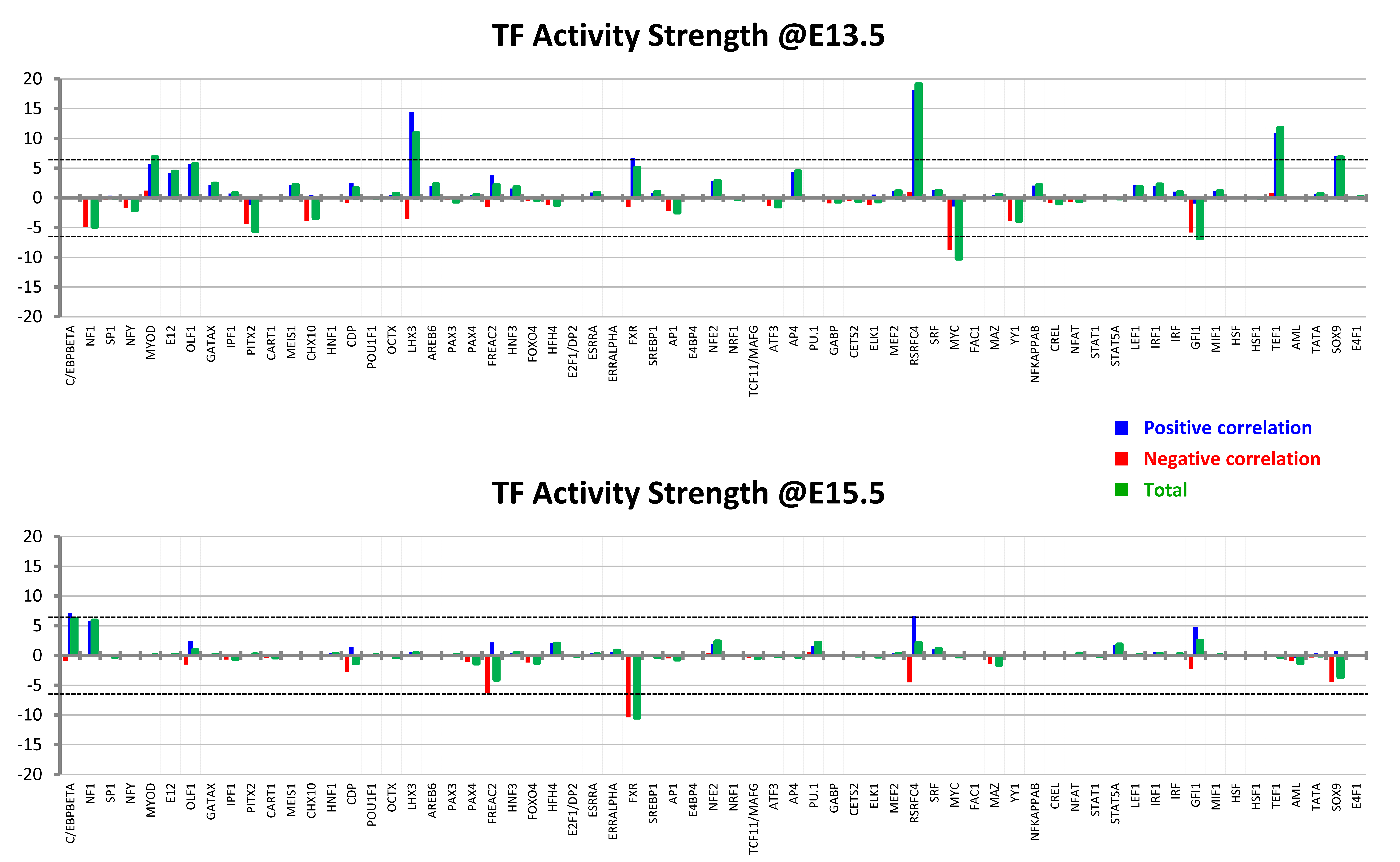

Supplement: Additional file 1: — Supplementary Text and Figures. (DOC 7227 kb) [file 12859_2017_1636_MOESM1_ESM.doc]
